# Osteoblastic and Bacterial Response of Hybrid Dental Implants

**DOI:** 10.3390/jfb14060321

**Published:** 2023-06-13

**Authors:** Daniel Robles, Aritza Brizuela, Manuel Fernández-Domínguez, Javier Gil

**Affiliations:** 1 Department of Translational Medicine, CEU San Pablo University, Urbanización Montepríncipe, 28925 Madrid, Spain; drobles@clinica.uemc.es; 2 Facultad de Odontología, Universidad Europea Miguel de Cervantes, C/del Padre Julio Chevalier 2, 47012 Valladolid, Spain; abrizuela@uemc.es; 3 Department of Oral and Maxillofacial Surgery, University Hospital Monteprincipe, University CEU San Pablo, Av. de Montepríncipe, s/n, 28668 Madrid, Spain; mfdominguez@hm.ceu.es; 4 Bioengineering Institute of Technology, Facultad de Medicina y Ciencias de la Salud, Universidad Internacional de Catalunya, Josep Trueta s/n. Sant Cugat del Vallés, 08195 Barcelona, Spain

**Keywords:** roughness, wettability, titanium, osteoblasts, peri-implantitis, bacteria

## Abstract

Bacterial infections in dental implants generate peri-implantitis disease that causes bone loss and the mobility of the dental implant. It is well known that specific ranges of roughness favor the proliferation of bacteria, and it is for this reason that new dental implants called hybrids have appeared. These implants have a smooth area in the coronal part and a rough surface in the apical part. The objective of this research is the physico-chemical characterization of the surface and the osteoblastic and microbiological behavior. One-hundred and eighty discs of titanium grade 3 with three different surfaces (smooth, smooth–rough, and completely rough) were studied. The roughness was determined by white light interferometry, and the wettability and surface energy by the sessile drop technique and the application of Owens and Wendt equations. Human osteoblast SaOS-2 was cultured to determine cell adhesion, proliferation, and differentiation. Microbiological studies were performed with two common bacterial strains in oral infection, *E. faecalis* and *S. gordonii*, at different times of culture. The roughness obtained for the smooth surface was Sa = 0.23 and for the rough surface it was 1.98 μm. The contact angles were more hydrophilic for the smooth surface (61.2°) than for the rough surface (76.1°). However, the surface energy was lower for the rough surface (22.70 mJ/m^2^) in both its dispersive and polar components than the smooth surface (41.77 mJ/m^2^). Cellular activity in adhesion, proliferation, and differentiation was much higher on rough surfaces than on smooth surfaces. After 6 h of incubation, the osteoblast number in rough surfaces was more than 32% higher in relation to the smooth surface. The cell area in smooth surfaces was higher than rough surfaces. The proliferation increased and the alkaline phosphatase presented a maximum after 14 days, with the mineral content of the cells being higher in rough surfaces. In addition, the rough surfaces showed greater bacterial proliferation at the times studied and in the two strains used. Hybrid implants sacrifice the good osteoblast behavior of the coronal part of the implant in order to obstruct bacterial adhesion. The following fact should be considered by clinicians: there is a possible loss of bone fixation when preventing peri-implantitis.

## 1. Introduction

Currently, the use of titanium dental implants for the prosthetic rehabilitation of patients with partial or total edentulism represents a high percentage of the treatments performed in dental clinics worldwide. The survival rate of dental implants in both cases (partial and total rehabilitations) is between 95% and 100% [1,2]. Since Branemark et al. [3] introduced implantology into the world of oral surgery and oral rehabilitation in 1969, implantology has evolved over recent decades in different fields such as manufacturing materials, connections, and surfaces, among others. New materials and improvements in surgical techniques in relation to the original protocols have not only reduced treatment times but have also increased indications even in cases that previously could have been considered a risk of failure, such as poor bone availability.

Some of the great advances that have made this possible are those concerning the surface of implants, both topographically and chemically, focused on improving BIC (Bone Implant Contact) and osseointegration. Osseointegration is defined as the intimate contact between the implant and the surrounding bone [4,5]. In clinical terms, it is defined as the stable maintenance of an implant in function within the bone [6].

It has been demonstrated that rough implants obtained by projection of abrasive particles, especially alumina particles between 200 and 400 μm in size at 5 bar pressure, produce a roughness Sa between 1 and 2 μm, which is optimal for osteoblastic adhesion, proliferation, and differentiation [4]. For this reason, most dental implants are rough and have topographies between these values. This particle projection causes a compressive residual surface stress on the dental implant, which results in a longer fatigue life of the dental implant due to the impossibility of crack initiation on the dental implant surface [5].

However, this rough topography, which favors bone formation and produces a good biological fixation of the implant, is not suitable for bacterial colonization. It is well known that roughness favors bacterial proliferation, which over time leads to the formation of biofilm, causing peri-implantitis.

Peri-implantitis is an inflammatory disease of the tissues surrounding dental implants producing loss of the bone that fixes them [4]. Derks et al. determined a prevalence of peri-implant disease around 22% in 4209 implants studied [5]. In addition, Derks et al., in Sweden, conducted a randomized cross-sectional analysis, finding that 45% of patients had peri-implantitis after 9 years of implant placement and determining a bone loss greater than 0.5 mm, although this decreases to 14.5 mm when assessing a bone loss greater than 2 mm [6].

Peri-implantitis is a disease characterized by inflammation and bacterial colonization, its etiology being very complex and influenced by many factors [7,8].

The factors that influence the presence and progress of the disease have been studied and defined in multiple studies. There are many studies that show that poor hygiene and poor plaque control by the patient, pre-existing periodontal disease, and smoking are clear risk factors for the onset and establishment of the disease. Other related factors such as diabetes, genetic factors, absence of minimal keratinized gingiva, limited bone availability, occlusal overload, bone overheating, titanium particles, micromovement, biocorrosion, or microscopy and implant surface characteristics may play an important role in the onset and development of the disease, but their influence has not yet been defined [8,9,10].

Hybrid implants have two types of surface finishes. The coronal part has machined titanium (smooth) and the apical part has a rough surface. The machined coronal part with very small roughness values hinders bacterial adhesion and therefore reduces biofilm formation. This is due to several factors, including decreased wettability. This smooth surface also causes a decrease in cell adhesion. However, the rough side favors bacterial colonization and the adhesion of osteoblastic cells, favoring high levels of bone tissue growth [11,12,13,14]. Hybrid implants have good behavior, marginal bone stability, and fewer biological complications [15].

Hybrid dental implants have recently started to be used as implants that prevent peri-implantitis. When a biofilm is formed, the smooth topography facilitates its cleaning or implantoplasty. Different companies are manufacturing this type of implant guaranteeing good osseointegration behavior. However, there are no publications on the properties of the surface, nor on the behavior of the osteoblastic cells or their microbiological behavior. This contribution is original and aims to make clinicians aware of the most basic properties for their assessment before the placement of a hybrid implant. Unfortunately, due to the absence of works with hybrid implants, we cannot compare the results obtained. This contribution aims to determine the physico-chemical properties of the hybrid implant surfaces and to determine the in vitro behavior of human osteoblastic cells as well as the bacterial proliferation of two types of strains that are common in oral infection processes.

## 2. Materials and Methods

### 2.1. Materials

Hybrid implants present two types of surfaces, the coronal part up to the third turn is a smooth surface and from the third turn it is a rough surface up to the apex of the dental implant. For surface characterization from physico-chemical, biological, and microbiological points of view, it is necessary to follow the international standards for each test. For this reason, 180 commercially pure grade 3 titanium discs were manufactured with three different treatments (Klockner Dental Implants, Escaldes Engordany, Andorra):

- Smooth surface with the same finish as the coronal part of the hybrid implant: The surface was polished for 15 min in a diamond powder suspension. It was washed with water and alcohol for 5 min and air dried.
- Rough surface: The disc has the same roughness as the apical area of the dental implant. The rough part is obtained by projection of alumina particles of an average size of 200 μm at a pressure of 2.5 bars. The projection from the gun is about 150 mm to the surface. The discs were subsequently washed with water and alcohol for 5 min and air dried.
- Mixed surface: Half of the disc surface is smooth and the other half is rough.

Sixty discs were used for each treatment. The surfaces of the discs underwent the same treatments as those given to dental implants.

### 2.2. Roughness

The evaluation of the surface roughness of the implants was carried out using white light interferometry equipment (model Wyco NT1100 Optical Profiling System, Veeco Instruments, Tucson, AZ, USA) in the Vertical Scanning Interferometry (VSI) model equipped with “Vision 32” analysis software. Twenty measurements were realized for each surface.

### 2.3. Wettability and Surface Energy

The wettability of the dental implant surface was determined using the sessile drop technique to determine the contact angle. This technique is based on observations by optical microscopy (Con-tact Angle System OCA15plus, Dataphysics, Germany) and determination of the angle with proprietary software (SCA20, Dataphysics, Riverside, CA, USA). Ten tests were performed for each surface at a temperature of 37 °C and 100% relative humidity, simulating physiological conditions. In addition, the static contact angles, CA, of two reference solutions were determined using the same method with pure water and at the same temperature and relative humidity conditions. Wettability was studied using a contact angle goniometer (OCA 15+, Dataphysics, Riverside, CA, USA).

The total surface free energy (SFE) was determined by the sum of the dispersive component, also called “London” and the “polar” component of all the samples studied. The SFE was calculated with the contact angles obtained with the three different solutions: ultrapure distilled water (MilliQ, Sigma Aldrich, St. Louis, MO, USA) and di-iodomethane. The values obtained were calculated using the Owens and Wendt equations.

(1) $$\gamma_{S}=\gamma_{SL}+\gamma_{L}\cos\theta$$

(2) γL1+cos⁡θ=2γLdγSd12+γLpγSp12

where *γ_S_* is the surface tension of the solid phase (*S*), *γ_L_* is the surface tension of the liquid (*L*), *γ_SL_* is the interfacial free energy or SE between *L* and *S*, *θ* is the contact angle between *L* and *S*, and *γ_d_* and *γ_p_* represent the dispersive and polar components of the SE, respectively [16,17,18,19].

### 2.4. Cell Viability and Differentiation

Cell assays were performed with osteoblasts (SaOS-2; ATCC, Manassas, VA, USA) using Dulbecco’s modified Eagle medium (DMEM) and McCoy’s modified 5A medium (Merck, Rahway, NJ, USA). To the media used, two solutions of 10% fetal bovine serum (FBS) and 50 µg/mL L-glutamine and penicillin/streptomycin at a concentration of 2 mM (Invitrogen, Carlsbad, CA, USA) were added. These cultures were developed at body temperature in an incubator with a 5% CO_2_ atmosphere and 100% humidity. For each treatment, 25 samples were cultured.

Separation of confluent cells was performed for 1 min using cultures incubated with TrypLE (Invitrogen, Carlsbad, CA, USA). The osteoblast dilution was centrifuged, and the culture medium was renewed. After this, the samples were seeded for each surface with 5000 cells for each of the discs studied and incubated at 37 °C. After 6 h of incubation, the discs were washed with phosphate-buffered solution (PBS) and transferred to a new plate to perform the metabolic activity assay using Alamar Blue (Invitrogen-Thermo Fisher Scientific, Waltham, MA, USA). The reagent was prepared, the samples were coated, and the percentage reduction of Alamar Blue was assessed after 6 h at 37 °C temperature. Alamar Blue was used as a blank.

To evaluate cell proliferation, cells were multiplied on the surface of the materials studied at different incubation times. In this study, it was evaluated at 6 h (adhesion time) and 3, 7, 14 and 21 days. Cell quantification was performed by quantifying the enzyme “lactate dehydrogenase, LDH”, which is produced in similar quantities in each cell. Knowing the amount of LDH released into the medium when lysing the cells, we can know the number of cells in a sample by extrapolation, the application of which requires the determination of a standard curve. The quantification of LDH was carried out using a Cytotoxicity Detection Kit (Roche, no. 11644793001). This kit uses a colorimetric chemical reaction that triggers a color change of LDH-containing suspensions proportional to the concentration of LDH. The color and/or rate of color change was quantified using a spectrophotometer (Synergy HTX, BioTek, Thermo Fisher, Waltham, MA, USA) at the wavelength of 492 nm. In order to enable cell quantification, a standard line was made by increasing numbers of cells to correlate the absorbance with the number of cells. The absorbance of the samples was measured using a spectrophotometer (Optical Density, Olympus, Tokyo, Japan) and correlated with the number of cells using the standard curve, in which the number of cells in the well is known in advance.

Cell differentiation assays allow assessment of the degree of cell specialization by means of specific markers. A significant indicator of the onset of osteoblastic differentiation is alkaline phosphatase, which is an enzyme indicating the onset of bone formation. The enzyme concentration was calculated using the “SensoLyte^®^ pNPP Alkaline Phosphatase Assay Kit *Colorimetric” with (ref. 72146, AnaSpec, Sigma Aldrich, Saint Louis, MO, USA). The same lysates from the proliferation assay were used. Following the kit instructions, the absorbance was measured at 405 nm and extrapolated to a standard line with purified alkaline phosphatase. The results were divided by the corresponding cell number (obtained in proliferation) and by the incubation time of the reaction (at 37 °C).

In addition to the analysis of cell differentiation by the ALP method, the determination of the level of bone mineralization was also carried out, which allows the quantification of the amount of calcium deposits that the cells have deposited on the tested surfaces after 21 days of incubation. Staining with “alizarin red S” was performed to identify the areas with calcium deposits. Images were taken with a microscope and then the stained deposits were extracted and quantified by a spectrophotometer (Synergy HTX, BioTek) at 570 nm.

### 2.5. Bacterial Adhesion

Two types of bacteria, *E. faecalis* (CECT 795) and *S. gordonii* (CECT 804), were studied for the microbiological characterization using tryptic soy broth (TSB) for *S. gordonii* and brain heart infusion (BHI) for the rest of them as the culture media. Five discs per group and bacterial strain were studied. These two bacterial strains were chosen because they are frequently found in peri-implantitis and are among the most pathogenic of the Gram + strains. These strains are more aggressive than *S. sanguinis* or *L. salivarius* which are also frequent in the literature [7].

Samples and culture media were autoclaved at 121 °C for 30 min. Sterilization of the titanium consisted of washing with ethylic alcohol for 300 s followed by three washes with water and application of ultraviolet light for 15 min to each surface.

Bacteria for the cultures were allowed to grow for 10 h in an oven at 37 °C, suspending the bacteria in 5 mL of the corresponding medium. The bacterial inocula were diluted until the optical density reached 600 nm. The sterilized discs with the different surfaces were placed in 24-well plates, coating the discs with 700 µL of the diluted bacterial solution, making sure to cover the entire surface of the sample. The discs were placed in an incubator at 37 °C for 2 h. The discs were prepared for the determination of bacterial adhesion. As a positive control, 700 µL of the bacterial suspension was added to an empty microwell plate. After this time, the samples were washed with PBS and transferred to a new 24-well plate for metabolic activity assays and determination of live/dead bacteria.

In order to determine the metabolic activity, five samples as well as the positive controls were placed in a 650 µL solution of 25 µg/mL resazurin sodium salt in PBS (Sigma-Aldrich, St. Louis, MO, USA) at body temperature until saturation of the positive control. An amount of 100 µL of each sample was extracted to determine the absorbance at 570 and 600 nm and the variations were calculated.

Three samples were stained with the LIVE/DEAD^®^ BackLight™ Bacterial Viability Kit (ThermoFisher Scientific, Waltham, MA, USA). Chemical reagents were diluted to a concentration of 1.5 µL of reagent per mL of PBS, covering 650 µL of the dilution. They were then placed in an incubator at body temperature for a quarter of an hour. Finally, three successive washes with PBS were performed and images were observed in three different areas with a confocal laser microscope at 64× magnification (Leica Dmi8, Wertzlar, Germany) using excitation/emission wavelengths of 495/520 nm for live cells and 589/615 nm for dead cells.

### 2.6. Statistical Analysis

The data were statistically analyzed using Student’s *t*-tests, a two-way ANOVA, and Turkey’s multiple comparison tests to evaluate any statistically significant differences between the sample at *p* < 0.005.

## 3. Results

**Figure 1:**
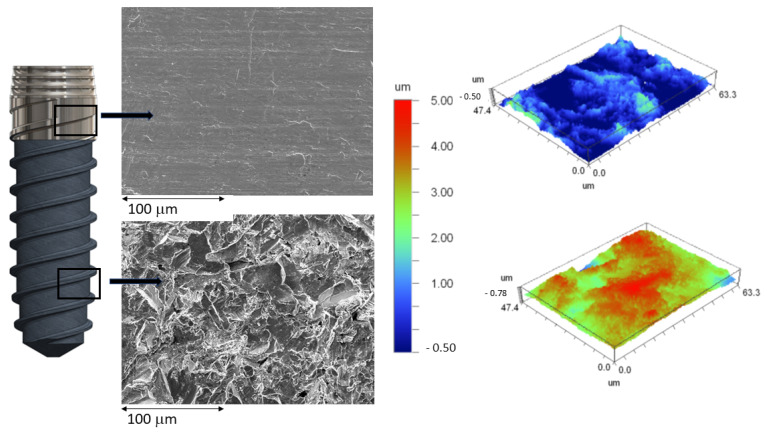
Microstructures of smooth and rough zones of the hybrid implant and their 3D topographic maps.

Figure 1 shows the topographies of the smooth and rough parts of the dental implant as well as the roughness maps obtained using the interferometry technique by scanning electron microscopy. The roughness results obtained are shown in Table 1.

The values of the roughness measured are illustrated in Table 1. The differences observed for Sa, Sm, and index area confirmed that the roughness values present statistically significant differences (*p* < 0.05).

Contact angles (CA) obtained with water and the results of the surface free energy (SFE) are shown in Table 2 and Table 3. Following the Owens and Wendt equations, the polar and dispersive energies can be determined [20,21,22,23]. In general, the abrasive projection decreased the wettability of the surface, i.e., increased the contact angle. This fact was especially pronounced on surfaces deformed with alumina particles.

Comparing the contributions of the dispersive and polar components of the surface energy, one can observe a decrease in the polar component for the samples with residual alumina (Table 2 and Table 3). Statistically significant differences were found in the polar component of the rough surfaces with alumina compared to the smooth ones.

**Figure 2:**
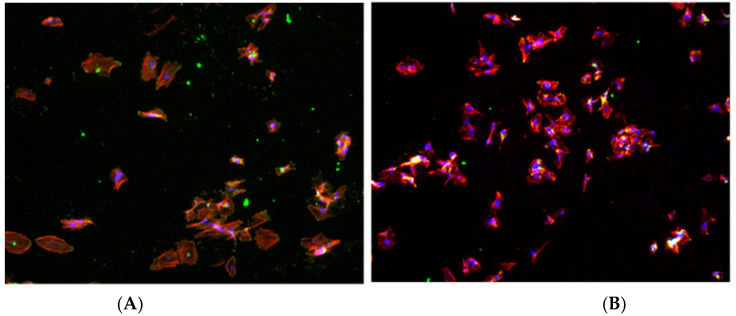
Fluorescence images of the samples studied. (**A**) Smooth surface. (**B**) Rough surface.

Figure 2 shows the images obtained by fluorescence microscopy, showing the cell nuclei in blue, the cell skeleton in red, and finally the focal points in green. In the image of the smooth zone (Sa = 0.23), the cell morphology is clearly polygonal, while in the rough zone (Sa = 1.98), the cells present a rather irregular shape.

**Figure 3:**
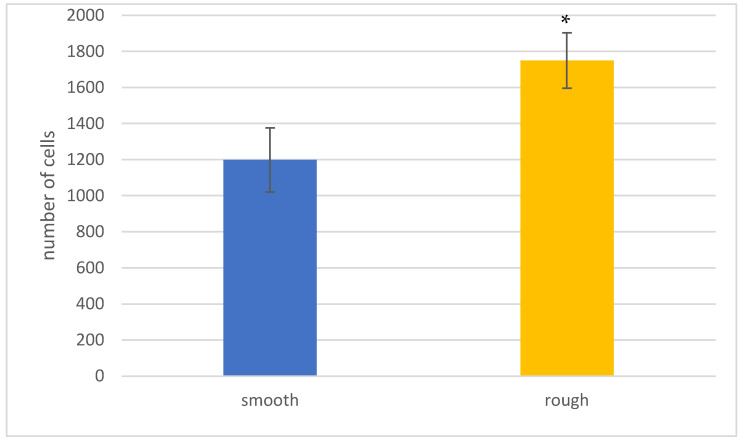
Number of osteoblasts after 6 h on each surface: smooth and rough. An asterisk means statistically significant differences.

**Figure 4:**
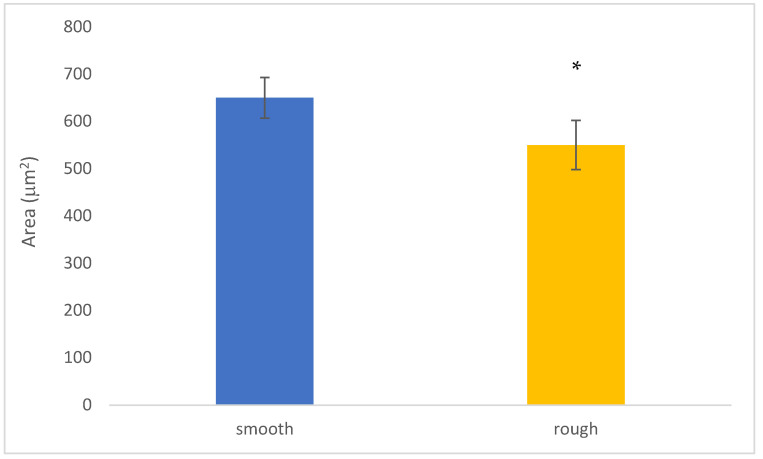
Cell area in m^2^ of osteoblasts after 6 h in each surface: smooth and rough. An asterisk means statistically significant differences.

Figure 3 shows that the cell behavior in the first hours of contact with the different samples allow discerning statistical differences in terms of cell adhesion behavior. The treatment of the images acquired by fluorescence microscopy has made it possible to determine the average area occupied by each cell, the results of which are presented in Figure 4.

In the experimental data presented in Figure 4, it is clearly observed that the cells have a larger area in the smooth samples compared to the shot-blasted samples. On the surface of the shot-blasted samples, the presence of cells scattered over the entire surface is observed, while in the smooth samples, the formation of cell clusters in specific areas of the surface is observed. However, the cell geometry and morphology are good in all the conditions studied, presenting an extended shape on the surface.

**Figure 5:**
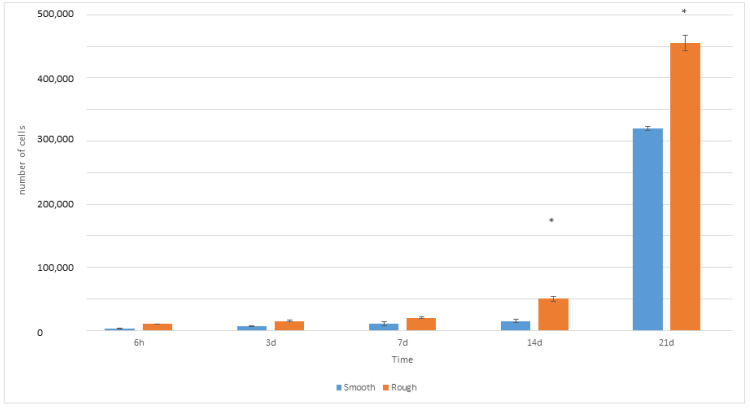
Proliferation at different times for each surface: smooth and rough. An asterisk means statistically significant differences.

The values of cell proliferation are shown in Figure 5. The analysis of the LDH quantification values reveals a progressive and generalized increase in cell proliferation during the whole culture time.

**Figure 6:**
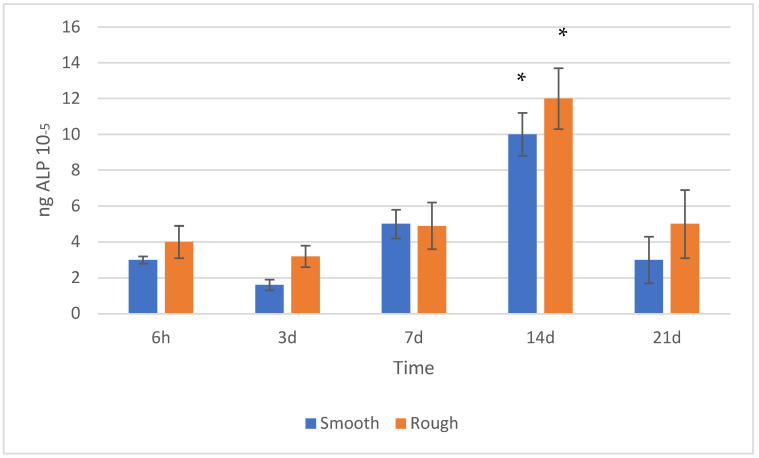
Alkaline phosphatase at different times for each surface: smooth and rough. An asterisk means statistically significant differences.

The results obtained in the cell proliferation differentiation assay can be observed in Figure 6.

**Figure 7:**
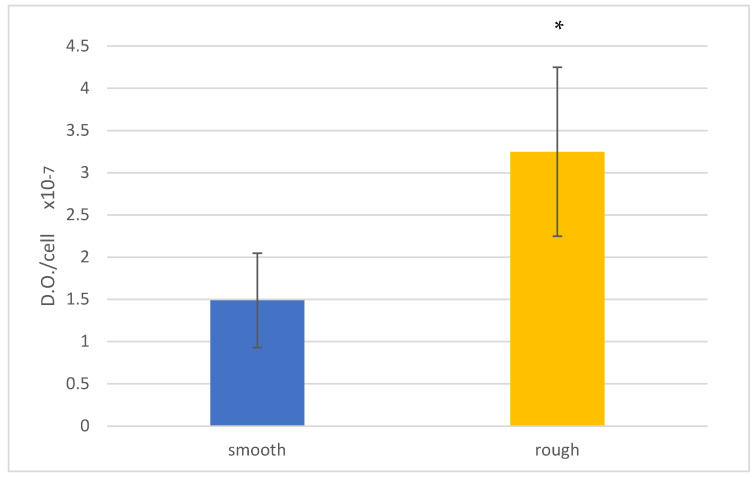
Mineral content of the cells after 21 days for each surface: smooth and rough. Asterisk means statistically significant differences.

The analysis of the values allows us to evaluate the degree of mineralization reached on each surface for the 21 days of culture. The analysis of the results allows observing a higher mineralization at 21 days of culture on the rough surface (Figure 7).

**Figure 8:**
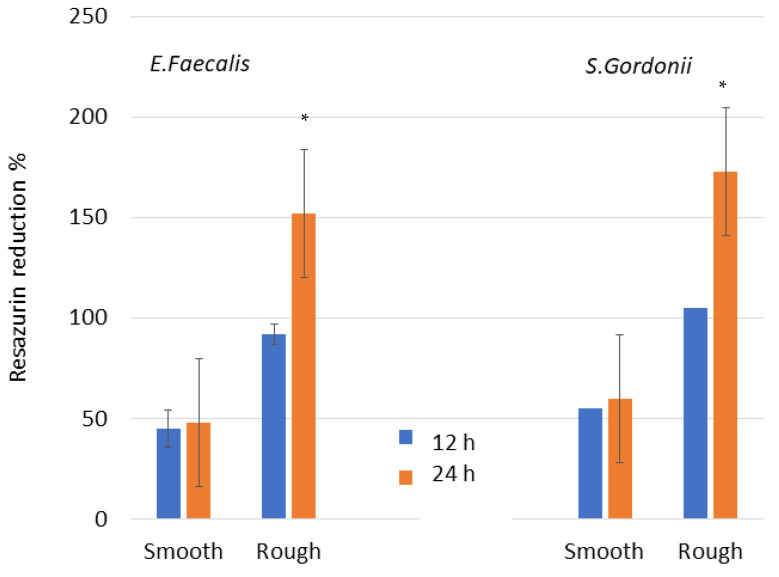
Metabolic activity assay of *E. faecalis*. Asterisk means statistically significant differences.

The values for the metabolism and Live/Dead test for each type of stain are illustrated in Figure 8. The bacteria metabolism results present significant differences between smooth and rough surfaces, with *p* < 0.005. Smooth discs have a lower bacterial adhesion in both surfaces and at different test times.

**Figure 9:**
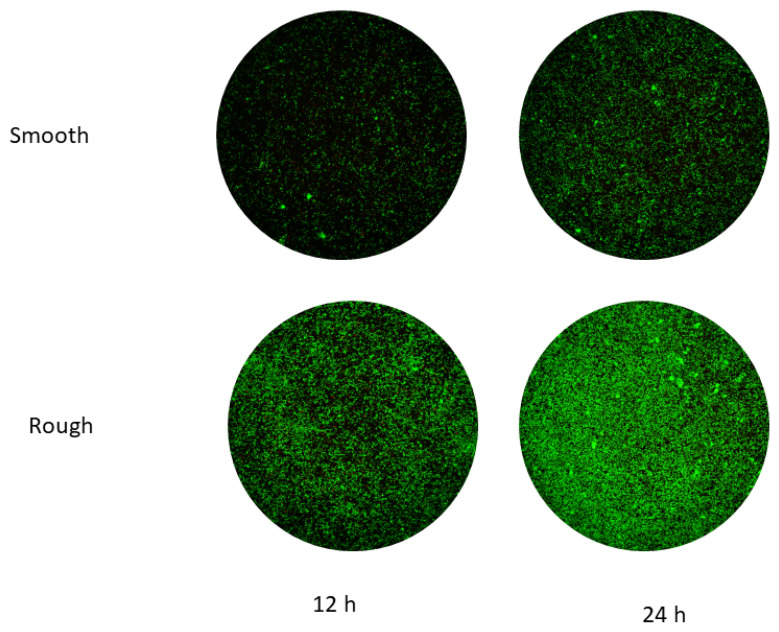
Live/Dead images of *E. faecalis*. A green color corresponds to alive cells and red to dead cells.

When observing the Live/Dead images, the results point at an important difference in the number of bacteria between both surfaces. For both surfaces, a growth in the number of bacteria is also observed between 12 and 24 h. Figure 9 shows the Live/Dead images for both surfaces and different times for the *E. faecalis* strain. These results agree with the metabolic activity results.

## 4. Discussion

We can observe that the roughness values generated by the abrasive projection generate a higher contact angle, i.e., the surface becomes more hydrophobic with respect to the smooth surface. In the same way, it can be observed that the surface energy decreases as the roughness increases, both in its polar and dispersive components.

The factors which play a key role in the biological activity on the surface of the biomaterials (cell adhesion and modulate cell–titanium interactions [24,25,26]) are the surface chemistry [27,28,29], wettability [30,31], and roughness [32,33]. Numerous research works have been conducted in order to understand the factors governing cellular adhesion to surfaces, but they are still limited [34,35,36]. However, some evidence has been demonstrated in relation to the cell–surface interactions. Firstly, the adsorption of fibronectin (Fn) and albumin (Alb) was studied at a wide range of wettabilities. A low contact angle favors Fn adsorption, while higher adsorption on hydrophobic surfaces was observed for Alb. By performing protein competition studies between Fn and Alb, it was observed that hydrophilic character produces a higher adsorption of Fn, whereas Alb is adsorbed on hydrophobic surfaces. The initial adhesion of osteoblastic cells increased with surface wettability, particularly on superhydrophilic surfaces; these results confirm the Fn adsorption in the competitive test. These studies suggest that Fn adsorption may be responsible for increased cell adhesion on hydrophilic surfaces in a body fluid or culture medium under physiological conditions. However, the surface energy leads to an increase in fibronectin adsorption, which would go against the trend towards hydrophilicity of the surface [37,38]. Another factor is roughness and its compressive surface tension, which favor cell adhesion, as has been demonstrated by several authors [39,40]. Therefore, on our surfaces, we have different adsorption tendencies in different directions, but what is clear from the results is the greater adhesion of human osteoblasts on rough surfaces. It could be said that the values of the roughness and the surface energy have a greater influence than the contact angle. Furthermore, we have to consider in our study that although the rough surface has a greater contact angle than the smooth surface, the difference is around 10°, which does not produce a great difference in the hydrophilicity of the surface. One should bear in mind that techniques have been studied, such as thermal treatments [41] or the introduction of hydroxyapatite particles to improve osteoblastic adhesion [42], which change the contact angle of surfaces with hydrophobic values of 100° to superhydrophilic values of around 0° [43]. In our case, the differences in contact angle are small and do not play a decisive role in osteoblastic adhesion.

The cell area results are higher for cells adhering to smooth surfaces and this may be explained by the limiting effect of the rough topography on osteoblast extension. However, it has been shown that osteoblastic cells on rough surfaces have a greater number of focal points and filopodia than those on smooth surfaces, which results in a greater adhesion force of the osteoblast on the surface [40,41,42,43,44].

It can be observed that the ALP signal increases progressively from the first day to day 14, and then decreases until day 21. This behavior in terms of cell differentiation is usual and is duly described in the scientific literature as a typical early cell differentiation process [45]. The inflection in the ALP signal level after a certain time of proliferation is related to this enzyme (ALP), which is an indicator of the onset of cell differentiation. A drop in phosphatase activity after a certain time is considered normal, since cells experience a peak in the generation of this enzyme during differentiation (specialization). Once the differentiation of the cells has taken place, the generation of alkaline phosphatase decreases and mineralization increases, which would explain the reduction observed between 14 and 21 days of culture. As for the comparison of the degree of cell differentiation as a function of surface roughness, the analysis allows us to identify a maximum ALP concentration at 14 days for the rough surface.

Microbiological studies have shown an increase in bacterial colonization of *E. faecalis* and *S. gordonii* strains in rough samples and with culture time. As is well known, roughness facilitates bacterial colonization, except for nanometer scale roughness, since it has a better accommodation. As has been studied, the residual surface tension caused by the projection of particles at high pressures to form the roughness facilitates colonization. This compressive stress, which according to the authors is approximately −200 MPa, allows the bacteria to establish a better adhesion with the titanium and in some cases bacterial corrosion has been determined due to this residual stress.

From the results obtained, it can be affirmed that the hybrid implants have two very specific zones with different functions. In the rough zone, we have been able to verify how adhesion, proliferation, and differentiation are superior compared to the smooth surface. This surface will have a higher level of osseointegration and will produce an increase in the biological fixation of the implant.

The smooth coronal surface does not have optimal properties for osteoblast cellular activity and therefore the levels of osseointegration in this area will be lower, as we have been able to verify from the results obtained. However, this area will hinder bacterial colonization as we have also been able to determine. This surface is in the coronal area close to the oral cavity where the infection will be provoked and then filter towards the body of the dental implant. A strategy in which the primary connection areas of the dental implant are polished will prevent or at least hinder bacterial colonization and the subsequent formation of a biofilm.

It should be taken into account that this strategy of surface polishing the coronal part of the dental implant to hinder mucositis and posterior peri-implantis is detrimental to the osseointegration of the dental implant. In other words, we sacrifice bone formation and reduce the bone index contact of the dental implant in order to have a less favorable surface for bacteria. Hybrid dental implants partially change the osseointegration by prevention if there is peri-implantitis. Hybrid implants can be an alternative since the number of failures due to peri-implantitis exceeds 20% in Europe and in the United States [46,47]. However, it would be better to maintain the optimal roughness of the dental implants to achieve the best bone values and to work on the crown of the implants and on the connecting abutments so that they have bactericidal products, such as silver nanoparticles, peptides such as Lactoferrin, or organic molecules such as TESPSA, which can have a bactericidal or at least bacteriostatic character, as can be the case of polyethylene glycol which can be functionalized on the surface of the titanium [48,49,50,51].

This contribution has the limitation that we have only worked with two bacterial strains and it should be carried out with biofilms to better observe the microbiological behavior. There is also the limitation of not having in vivo results that could shed more light on the results as well as validate all the in vitro studies performed. Another limitation is that the recent placement of dental implants does not allow for long-term clinical studies to determine the osseointegration behavior of dental implants, as well as the resistance to bacterial colonization [52,53,54]. Anyway, the studies clearly show the biological and microbiological behavior, and the advantages and disadvantages of the new hybrid implants can be appreciated so that clinicians can have more information for the selection of the best dental implants for their patients.

## 5. Conclusions

It has been determined that the smooth area of the coronal part of dental implants is more hydrophilic than the rough part but has a higher surface energy. The rough samples present a greater number of cells adhered to the surface after 6 h of culture, showing a smaller extension area on the surface than the smooth surfaces. The proliferation and mineral content of osteoblasts on the rough surface are higher than on the smooth surface. However, the smooth surface offers the least amount of bacterial adhesion in the studied strains (*E. faecalis* and *S. gordonii*) at different times, demonstrating that the surface performs better against bacterial proliferation.

## Figures and Tables

**Table 1:** Titanium surface roughness.

Surface	Sa (µm) ± SD	Sm (µm) ± SD	Index Area ± SD
Smooth	0.23 ± 0.02	0.33 ± 0.01	1.10 ± 0.02
Rough	1.98 ± 0.12 *	5.40 ± 0.20 *	1.16 ± 0.05 *

* Symbols indicate differences in relation to the two surfaces with *p* < 0.05.

**Table 2:** Contact angles for the different dissolutions used on the different surfaces.

Surface	Water CA’ (°)	Di-iodomethane CA’ (°)	Formamide CA’ (°)
Smooth	61.2 ± 0.6	50.6 ± = 0.9	50.8 ± 1.0
Rough	76.1 ± 0.3 *	63.2 ± 1.4 *	57.7 ± 1.0 *

* Statistical differences in each column are indicated by asterisk symbol (*p* < 0.05).

**Table 3:** Surface energy and its dispersive and polar components.

Surface	Surface Energy (mJ/m^2^)		
	Total	Dispersive Component	Polar Component
Smooth	41.1 ± 3.2	24.7 ± 3.2	16.4 ± 4.0
Rough	27.7 ± 1.8 *	18.7 ± 1.1 *	9.0 ± 3.5 *

* Statistical differences in each column are indicated by an asterisk symbol (*p* < 0.05).

## Data Availability

The data that support the findings of this study are available from the corresponding author upon reasonable request. The data are not publicly available due to possible to technological transfer.
